# Animal Models, Pathogenesis, and Potential Treatment of Thoracic Aortic Aneurysm

**DOI:** 10.3390/ijms25020901

**Published:** 2024-01-11

**Authors:** Yutang Wang, Indu S. Panicker, Jack Anesi, Owen Sargisson, Benjamin Atchison, Andreas J. R. Habenicht

**Affiliations:** 1 Discipline of Life Science, Institute of Innovation, Science and Sustainability, Federation University Australia, Ballarat, VIC 3353, Australia; indu.p@federation.edu.au (I.S.P.);; 2 Institute for Cardiovascular Prevention, Ludwig-Maximilians-Universität München (LMU), 80336 Munich, Germany; andreas.habenicht@med.uni-muenchen.de

**Keywords:** Marfan syndrome, β-aminopropionitrile, calcium chloride, elastase, angiotensin II

## Abstract

Thoracic aortic aneurysm (TAA) has a prevalence of 0.16–0.34% and an incidence of 7.6 per 100,000 person-years, accounting for 1–2% of all deaths in Western countries. Currently, no effective pharmacological therapies have been identified to slow TAA development and prevent TAA rupture. Large TAAs are treated with open surgical repair and less invasive thoracic endovascular aortic repair, both of which have high perioperative mortality risk. Therefore, there is an urgent medical need to identify the cellular and molecular mechanisms underlying TAA development and rupture to develop new therapies. In this review, we summarize animal TAA models including recent developments in porcine and zebrafish models: porcine models can assess new therapeutic devices or intervention strategies in a large mammal and zebrafish models can employ large-scale small-molecule suppressor screening in microwells. The second part of the review covers current views of TAA pathogenesis, derived from recent studies using these animal models, with a focus on the roles of the transforming growth factor-beta (TGFβ) pathway and the vascular smooth muscle cell (VSMC)-elastin-contractile unit. The last part discusses TAA treatment options as they emerge from recent preclinical studies.

## 1. Introduction

Thoracic aortic aneurysms (TAAs) refer to a localized thoracic aortic dilatation of greater than 50% of the normal diameter. TAAs can occur in the ascending aorta, the aortic arch, and the descending thoracic aorta, with some aneurysms involving multiple segments. The majority of TAAs involve the ascending thoracic aorta (60%), followed by the descending aorta (40%) and the aortic arch (10%) [1,2]. TAAs have an estimated prevalence of 0.16–0.34% and an incidence of 7.6 per 100,000 person-years [3]. TAAs account for 1–2% of all deaths in Western countries [4]. 

Risk factors for TAA development include male sex, older age, smoking, hypertension, chronic obstructive pulmonary disease, coronary artery disease, previous aortic dissection, and a family history of TAA and TAA-related disorders including bicuspid aortic valves, Ehlers–Danlos syndrome, Loeys–Dietz syndrome, Marfan syndrome, and Turner syndrome [5,6]. Atherosclerosis may be a risk factor for TAA [7]. It has been reported that atherosclerosis negatively affects the biomechanical properties of the descending thoracic aorta and decreases aortic resistance to tearing [8]. The decrease in aortic resistance to tearing strongly correlates with the presence of calcification [8]. It is worth noting that a recent study shows that TAA is not associated with atherosclerotic burden [9]. In addition, statin use is not associated with long-term mortality in patients with TAA undergoing endovascular repair [10].

There are two aortic repair-type interventions to treat large TAAs: open surgical repair and thoracic endovascular aortic repair (TEVAR) [11]. For treating TAAs in the descending thoracic aorta, open surgical repair is associated with a high perioperative mortality risk ranging from 2.7–8%, and the risk for TEVAR ranges from 2.1% to 6.1% [11,12,13]. Meta-analyses of non-randomized comparison studies have shown that early mortality is lower after TEVAR than open surgical repair [11,12,13,14,15]; however, overall mid-term survival (≥1 year) does not differ between these two treatment options [12,14]. In addition, endoleaks occur in 8.1% to 19.5% of cases after TEVAR [14,15,16]. In patients involving the ascending aorta (e.g., those with Marfan syndrome), open surgical repair should be preferred over TEVAR [17,18] as TEVAR is associated with a higher mortality rate of 15.2% [19].

Small TAAs are monitored by repeated computed tomography (CT) or magnetic resonance imaging (MRI). Once the 5.5 cm threshold is reached, patients without risk factors for dissection will be referred to aortic repair procedures [1,20]. 

Patients with TAAs are recommended to lower blood pressure using β-blockers, angiotensin-converting enzyme inhibitors, and angiotensin receptor blockers, to potentially reduce rupture and dissection risk [1]. However, no effective pharmacological therapies have been identified to slow TAA development and prevent rupture. 

Considering the potential limitations of open surgical repair and TEVAR and the lack of effective pharmacological therapies for TAA, it is a major medical need to understand the cellular and molecular mechanisms underlying TAA pathogenesis better and to develop new therapies including those to prevent endoleak following TEVAR. This review focuses on animal TAA models, and recent developments in disease pathogenesis and potential TAA treatments derived from studies using these animal models.

## 2. Pathological Features of Human TAAs

The aorta is composed of three layers: the tunica intima, tunica media, and tunica adventitia. The tunica intima is composed of one layer of endothelial cells attached to the basal lamina. The tunica media is composed of more than 50 alternating layers of vascular smooth muscle cells (VSMCs) and elastic fibers. The tunica adventitia is composed of fibroblasts, loose connective tissue, and vasa vasorum [21]. The major pathological features of human TAA include endothelial dysfunction, elastin fragmentation, loss of VSMCs via increased apoptosis, increased deposition of proteoglycans in the tunica media, and excessive accumulation of collagen (vascular fibrosis) [21,22,23,24]. These changes are often accompanied by an increase in inflammation, oxidative stress, and matrix metalloproteinases (MMPs) [23].

TAAs become progressively larger and this enlargement enhances the risk of aortic dissection and rupture [21]. Thoracic aortic dissections develop when a tear in the tunica intima occurs, which allows blood to flow into the aortic wall to form a false lumen. 

## 3. Rodent TAA Models

Rodents are the most commonly used animals in TAA research and they have contributed greatly to our understanding of the pathogenesis of this disease. TAAs in rodents can be induced by various methods including chemicals, surgery, and genetic manipulation.

### 3.1. β-Aminopropionitrile (BAPN)-Induced TAA in Rodents

BAPN is the most commonly used chemical to induce TAAs in rodents [25,26,27,28,29,30,31,32,33,34,35,36,37,38,39,40,41,42,43]. BAPN is an inhibitor of lysyl oxidase, and the latter mediates the crosslinking process of elastin and collagen. Therefore, BAPN treatment weakens the arterial wall and promotes aneurysm formation [44,45].

Often, 3-week-old male C57BL/6 mice are used in this model. Mice are treated with BAPN (1 g/kg body weight per day) via drinking water for 4 weeks to induce TAAs (Table 1). There are some variations in this model, including genetic background, age, and sex. For example, C57BL/10 mice [34], mice aged 4 weeks [41], and mice of both sexes [37] have been used. In addition, lower doses of BAPN (e.g., 0.5 g/kg body weight per day [34,35,36]) have been employed. Moreover, the induction time can be shortened to 1 or 2 weeks to observe early changes in TAA initiation [37] or it can be extended up to 26 weeks to observe late changes in disease progression including TAA rupture and mortality [41]. It is worth noting that rats are also used in this model [43] (Table 1).

TAAs induced by BAPN show thrombosis, degradation of elastic fibers, and loss of VSMCs [46], bearing similarities to human TAAs. In addition, this rodent model shows thoracic aortic dissection and therefore it can be used to investigate this TAA-associated complication. 

### 3.2. Angiotensin II Infusion-Induced TAA in Rodents

Angiotensin II is a commonly used TAA-inducer [47,48,49,50,51,52,53] (Table 2). In this model, apolipoprotein E-deficient mice are often employed [47,48,49]. To induce TAAs, mice are subcutaneously infused with angiotensin II at a dose of 1 μg/kg body weight per minute using an osmotic pump. Occasionally, higher doses of angiotensin II (e.g., 1.5 μg/kg body weight per minute [51]) are used. Angiotensin II-infused wild-type mice serve as controls to examine the impact of a certain gene on TAA formation [50,51,52]. Moreover, rats can be used in this model [53]. It is worth noting that angiotensin II infusion also induces aortic aneurysms in the abdominal region [54,55], and therefore, this model is a model of thoracoabdominal aortic aneurysms [56,57]. Finally, this model is also suitable for investigating TAA rupture and dissection.

### 3.3. Elastase-Induced TAA in Rodents

Elastase is another common TAA-inducer [58,59,60,61] (Table 3). Elastase is applied to the surface of the aorta using a presoaked sponge for a certain time [58,59,60,61]. Elastase can be applied to the ascending aorta and aortic arch [58] or the descending thoracic aorta [59,60,61]. Both adult mice [58,59,60] and rats [61] have been studied for this purpose. The elastase dose and application time vary and a dose-response curve is recommended for each new bottle of elastase as the digestive power of elastase differs from bottle to bottle [60]. An optimal application time would create an aortic expansion of 100–130% at the end of the experiment [60]. Longer elastase application time leads to TAA rupture [58].

An advantage of this model is that TAAs are formed in a short time window, often 2 weeks. In addition, the TAA location can be controlled. This model has some disadvantages. First, it may not closely represent human TAA development which requires years or decades. Second, elastase-induced aneurysms begin to decrease in size when they reach maximal dilatation at two weeks post-surgery, which is different from human TAAs that become progressively larger [60]. 

### 3.4. Calcium Chloride-Induced TAA in Rodents

Calcium chloride is also a common TAA-inducer [62,63,64,65] (Table 4). In this model, the descending thoracic aorta is treated perivascularly with calcium chloride (0.5 M) for 15 min with a presoaked gauze applicator [62,63,64,65]. This method has been used for both mice [62,63] and rats [64,65]. This animal model represents some features of human TAAs including an increase in apoptosis [64,65], inflammation [65], and extracellular matrix degradation [62,63]. 

However, this method usually generates a smaller increase in aortic diameter of about 18–25% [62,64], although a larger increase in aortic diameter (~60%) has also been reported [63]. Calcium phosphate treatment can increase the abdominal aortic diameter to a larger extent (95%) [66,67], and whether calcium phosphate is superior to calcium chloride for TAA induction remains to be investigated. It is worth noting that the TAA size does not further expand from week 4 to week 16 after the calcium chloride application. Therefore, this model is not suitable for investigating TAA progression and rupture.

### 3.5. Combination of BAPN and Angiotensin II-Induced TAA in Rodents

The combination of BAPN and angiotensin II has been reported to induce TAA [46,68,69,70] (Table 5). Often 3-week-old male C57BL/6 mice are used. Mice are treated with BAPN for 4 weeks followed by 1–3 days of angiotensin II infusion (1 μg/kg body weight per minute) to induce TAAs [46,68,70]. BAPN can be administrated via intraperitoneal injection [68], drinking water [69,70], or as a diet supplement [46]. Angiotensin II infusion can be extended to 28 days which increases the percentage of mice that develop TAA [68]. Angiotensin II infusion can also be administrated at the same time as BAPN [69]. This animal model shows a sex phenotype with female mice showing a smaller aortic expansion and less medial degradation compared with their male counterparts [69]. Moreover, aortic dissection may be studied [46]. Similar to human TAAs, the TAAs in these mice show intramural hematomas, elastic fiber degradation, and inflammatory cell infiltration [69].

### 3.6. Combination of High-Fat Diet and Angiotensin II-Induced TAA in Rodents

TAAs can be induced in C57BL/6 mice by feeding the animals with a high-fat diet for 8 weeks followed by subcutaneous infusion of angiotensin II (2 μg/kg body weight per minute) during the last 4 weeks [71]. The approach is suitable to study TAA-associated dissection, as more than half of the animals develop the phenotype [71].

### 3.7. Transverse Aortic Constriction-Induced TAA in Rodents

**Figure 1:**
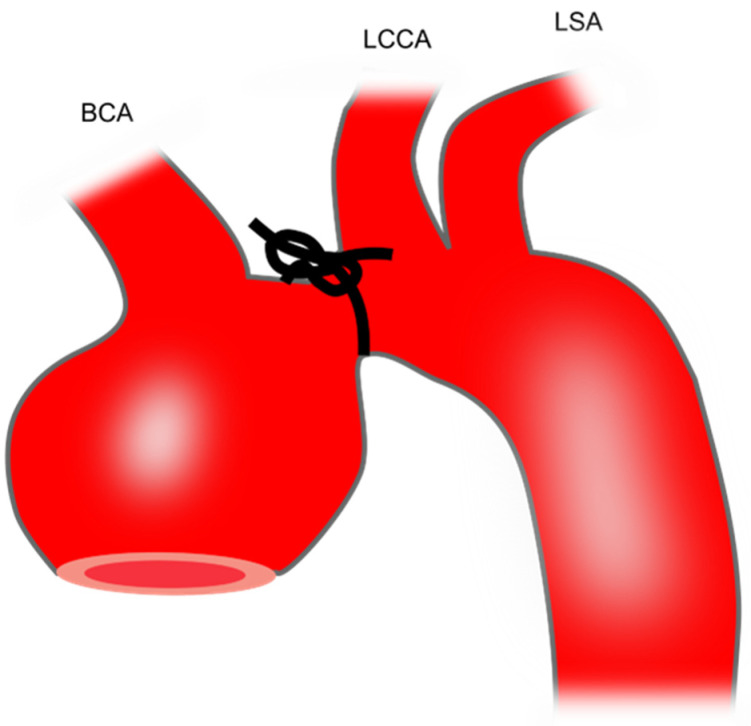
Preparation of mouse TAA using transverse aortic constriction. The black knot shows the area of constriction. BCA, brachiocephalic artery; LCCA, left common carotid artery; LSA, left subclavian artery; TAA, thoracic aortic aneurysm.

Transverse aortic constriction induces aortic dilation due to pressure overload. To perform this procedure, a silk suture (size: 6–0 or 7–0) is tied around a 27-gauge needle overlying the arch at a location between the brachiocephalic trunk and the left common carotid artery [40] (Figure 1). Then, the needle is promptly removed which yields a constriction of approximately 0.3 mm as the outer diameter of the 27-gauge needle. This produces aortic constriction of 60–80% [72]. 

This model leads to an increase in ascending aortic diameter by 21–23% after 1 [73], 2 [72], or 3 weeks [40]. Therefore, this model is not technically classified as a TAA model which requires an increase in aortic diameter of at least 50%. Whether the aortic diameter further increases with longer follow-up requires future study.

### 3.8. Genetic TAA Models in Rodents

Marfan syndrome affects 1 in 5000 individuals worldwide [74] and TAA (in particular at the aortic root and ascending aorta) is one of its clinical presentations. The disease is most commonly caused by a variant in the *Fbn1* gene which codes the fibrillin-1 protein. Fibrillin-1 interacts with elastin to provide strength and elasticity to blood vessels. Fibulin-4 (coded by the *Fbln4* gene) is important for elastogenesis. Therefore, defects in fibrillin-1 or fibulin-4 weaken the aortic structure and lead to TAA formation. The most commonly used genetic models of TAA are mice deficient in *Fbn1* or *Fbln4*. In addition, mice deficient in transforming growth factor-beta (TGFβ) receptor 2 are often used as a TAA model.

***Fbn1*^C1041G/+^ mice**: This mouse model of Marfan syndrome represents the most commonly used genetic model in investigating TAA [40,52,75,76,77,78,79,80,81,82,83,84,85,86,87]. *Fbn1*^C1041G/+^ mice, also known as *Fbn1*^C1039G/+^ mice, are generated by substitution of cysteine with glycine at amino acid 1041 (C1041G) in exon 25 of fibrillin-1 (previously identified in the literature as C1039G) [88]. This missense mutation presents in a subgroup of patients with Marfan syndrome [88]. These mice show a decreased deposition of microfibrils. The aorta of the *Fbn1*^C1041G/+^ mice starts to deteriorate after 2 months, showing overexpression of MMP-2 and -9, elastic fiber fragmentation, disarray of VSMCs, and increased collagen deposition [88]. Inflammatory cell infiltration is not prominent in this model. *Fbn1*^C1041G/+^ mice exhibit moderate TAA without dissection [88], have a normal lifespan [88], and show sex dependency, i.e., TAAs present dominantly in male mice [76,82].

***Fbn1*^mgR/mgR^ mice**: These mice are another model of Marfan syndrome and are the second most commonly used genetic model in TAA [49,89,90,91,92,93,94,95,96]. The *Fbn1* mgR allele is generated by insertion of the PGKneo expression cassette into an intron region of the *Fbn1* gene without loss of the *Fbn1* exon sequence [97]. The resultant mgR protein has the same size as wild-type fibrillin-1. *Fbn1*^mgR/mgR^ mice have reduced mgR expression of approximately 20% of the normal amount. These *Fbn1* hypomorphic mice rapidly develop ascending aortic aneurysms with macrophage infiltration, calcified tunica media, and elastic fiber fragmentation [97]. TAAs progress fast and dissection is fully penetrant in these mice. Therefore, this mouse line allows to investigate the progression of TAA-associated dissection and survival [97]. *Fbn1*^mgR/mgR^ mice represent a progressively severe model of Marfan syndrome and most of the affected animals die from dissecting TAAs within 3 months after birth [97,98]. 

***Fbln4*^SMKO^ mice**: Mice lacking fibulin-4 in smooth muscle cells (*Fbln4*^SMKO^) are another genetic model of TAA [49,99,100,101,102,103]. Animals die spontaneously when they are approximately 2 months old. Mice develop large aneurysms exclusively in the ascending aorta which is associated with a VSMC differentiation defect and focal hyperproliferation of VSMCs [104]. 

***Fbln4*^R/R^ mice**: The fibulin-4 R allele is generated by inserting a neomycin resistance gene-expressing cassette into the fibulin-4 gene, which leads to a 4-fold decrease in fibulin-4 expression through transcriptional interference [105]. All newborn mice develop TAAs at the ascending aorta resulting from disorganized elastic fiber networks. These mice start to die after 9 days. 

***Fbln4*^E57K/E57K^ mice**: The knock-in mutant mice are generated by substitution of glutamic acid with lysine at amino acid 57 (E57K) in exon 4 of the *Fbln-4* gene. The mutant fibulin-4 protein is prone to dimerization and is ineffectively secreted. Homozygous *Fbln4*^E57K/E57K^ mice can survive to 1 year of age and develop large TAA at the aortic root and ascending aorta (at least 2-fold increase in diameter compared with wild-type mice) in ~50% of the mice by 7 months [106,107]. 

***Tgfbr2*^SMKO^ mice**: These mice represent a conditional deletion of TGFβ receptor 2, specifically in smooth muscle cells. *Tgfbr2*^SMKO^ mice form dissecting TAAs in the ascending aorta and the aortic arch. Aortas show increased inflammation, elastic fiber fragmentation [108], and mural hematomas [109]. *Tgfbr2*^SMKO^ mice can cross-breed with *Fbn1*^C1041G/+^ to generate compound mutant mice (***Tgfbr2*^SMKO^/*Fbn1*^C1041G/+^ mice**) that have faster and more severe TAA growth compared with *Fbn1*^C1041G/+^ mice [109]. 

Other genetically modified mice can spontaneously develop TAAs and could be potentially used as genetic TAA models. For example, α-L-iduronidase-deficient mice (***Idua*^−/−^ mice**) display progressive accumulation of glycosaminoglycans in the aorta [110] and develop TAAs which are more severe in males than females [111,112,113]. ***Fbn1*^GT−8/+^ mice,** expressing a truncated fibrillin-1 protein display limited dilatation (<50%) of the thoracic aorta at the age of 8–12 months [114].

## 4. Porcine TAA Models

Although rodent models are very important experimental tools, there are differences between rodents and humans in metabolism, anatomy, and physiology [115]. In contrast, pigs better resemble human anatomy and physiology. For example, pigs and humans have similar heart rate and blood pressure [116,117,118,119,120]. 

Porcine TAA models have other advantages. They enable evaluation of new treatments for TAA by angiographic imaging, resembling clinical settings. In contrast, rodent models of TAAs are not suitable to be monitored using angiographic imaging. In addition, these porcine models could be used to assess the effectiveness of new therapeutic devices or interventions intended for clinical use in humans, in particular, to identify treatments for endoleaks after TEVAR [121,122] and new methods to minimize the damage caused by open surgical repair [123]. In contrast, rodent models are not suitable for testing TAA surgical repair procedures or devices due to the small size of the animals. 

One common limitation of using pig models, however, is that there is no reliable method to assess anesthetic depth during surgery [124].

### 4.1. Intra-Adventitial Injection of Elastase

**Figure 2:**
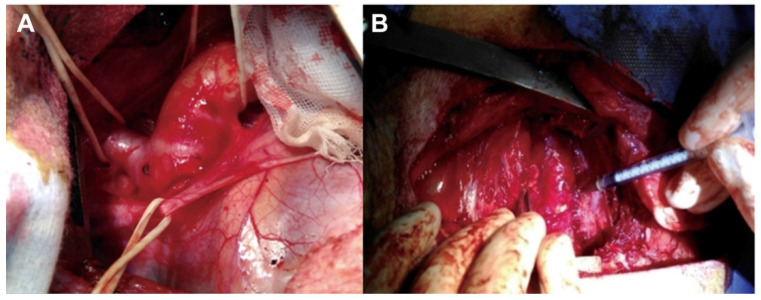
Preparation of porcine TAA using intra-adventitial elastase injection. (**A**), A 4 cm thoracic descending aortic segment proximal to the left subclavian artery is isolated. (**B**), Elastase is circumferentially injected into the tunica adventitia of the isolated segment by a bent 26-gauge syringe. Reprinted with permission from [18]. Copyright year, 2018, with permission from Elsevier.

Following thoracotomy, a 4 cm thoracic descending aortic segment proximal to the left subclavian artery is isolated. Elastase (a total of 5 mL, 20 mg/mL) is circumferentially injected into the adventitia of the isolated segment of the aortic wall starting from 0.5 cm away from the left subclavian artery and expanding 2 cm distally toward the diaphragm [18]. Twelve injection points are distributed in this 2 cm aortic segment with each point being injected with 0.4 mL of elastase [18] (Figure 2). This model is characterized by a loss of VSMCs and degradation of elastic fibers [18].

### 4.2. Intra-Adventitial Injections of Collagenase in Combination with Periadventitial Application of Calcium Chloride

Following thoracotomy, the descending thoracic aorta is dissected from the surrounding tissue to create a ~5 cm area [125]. Collagenase (5 mL, 0.35 mg/mL in saline with 0.1 mol/L calcium chloride) is circumferentially injected into the tunica adventitia of the isolated region. A piece of absorbable gelatin sponge is placed under the aortic region and 0.5 g of calcium chloride powder is then applied periadventitially to the isolated aortic segment. A gel foam is then wrapped around the aorta to enclose the area of calcium chloride application and to minimize irritation to the lungs and heart.

Three weeks after TAA induction, the aorta dilatates to 38 ± 13% without rupture. The aortas of these pigs show an increase in fibroblasts, an increase in MMPs, and a decrease in VSMCs [125].

### 4.3. Vein Patch Method

**Figure 3:**
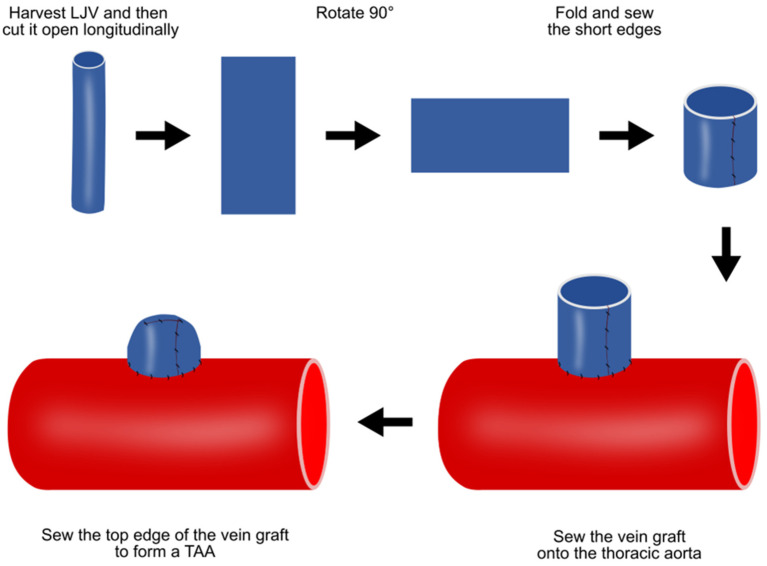
Preparation of porcine TAA using the vein patch method. LJV, left jugular vein; TAA, thoracic aortic aneurysm.

This vein patch method requires an invasive step for harvesting patch materials [16]. The left jugular vein is harvested with the maximum possible length and cut open longitudinally, and the two short ends are then sewn together (Figure 3). After thoracotomy, the exposed thoracic aorta is dissected from the surrounding tissue. The aorta is side-clamped and incised longitudinally for approximately three quarters of its diameter. The vein graft is then sutured to the aorta and the top line of the vein graft is sewn (Figure 3).

This method has several limitations. TAAs formed via this method are histologically different from real aneurysms. In addition, TAAs are saccular, not in a fusion form. However, this TAA model is expected to be valuable in developing novel treatments for endoleaks after TEVAR.

### 4.4. Pericardium Pouch Method

**Figure 4:**
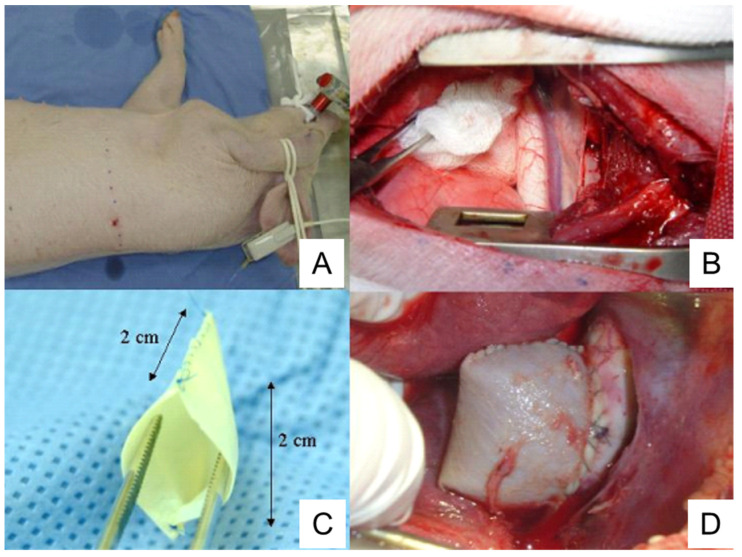
Preparation of porcine TAA using a pericardium pouch. (**A**), Pig positioning. The animal is laid on their right lateral side. (**B**), Exposure of thoracic aorta following thoracotomy. (**C**), Bovine pericardium pouch. (**D**), Aneurysm sac after the clamps are released. TAA, thoracic aortic aneurysm. The image is from [126], which was published under the terms of the Creative Commons CC BY 4.0 DEED (https://creativecommons.org/licenses/by/4.0/, accessed on 1 November 2023).

The commercially available bovine pericardium patch is treated with glutaraldehyde and conserved in 4% formaldehyde, treatment which provides proper characteristics to the patch including resistance, flexibility, and lack of antigenicity. The bovine pericardium patch is sewn on the lateral edges to form a 2 cm × 2 cm pouch structure (Figure 4). Following thoracotomy, the exposed descending thoracic aorta is clamped and a 2 cm aortotomy is created. Then, the pericardium pouch is sewn onto the aorta [126] (Figure 4).

Complete endothelization of the aneurysm sac is observed in 50% of animals. Mural thrombi are observed in 80% of animals [126]. An intense healing reaction with myofibroblasts occurs in the periadventitial region, which is not observed in human TAAs [126].

One advantage of the pericardium pouch method is that it can be used to train surgeons and develop new endovascular devices [126], as pigs exhibit anatomic and histopathological characteristics similar to human TAAs. TAAs formed by this method have some characteristics similar to human TAAs, such as the presence of mural thrombi and increased inflammation.

### 4.5. Cover-Then-Cut Method

**Figure 5:**
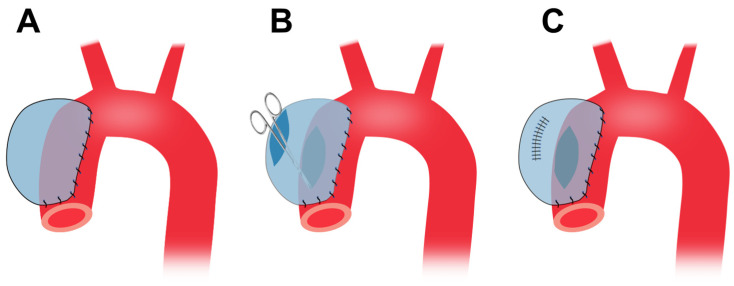
Preparation of porcine TAA using the cover-then-cut method. (**A**), The commercially available bovine pericardium patch is sewn onto the ascending aortic walls. (**B**), Following the side clamping, a large hole is made in the aortic wall which connects the patch cavity with the aortic lumen. (**C**), The patch opening is sutured. TAA, thoracic aortic aneurysm.

A piece of bovine pericardium, the same as the one described in Section 4.4, is tailored into a ~3.5 cm × 4.5 cm oval patch. Following thoracotomy, the porcine ascending aorta is isolated and the pre-prepared patch is sutured to the anterior and bilateral walls (Figure 5A). A longitudinal incision, ~3 cm in length, is made in the anterior wall of the patch. The aortic wall is side-clamped and a hole is made on the aortic wall by cutting away some tissue underneath the patch which connects the pericardial patch cavity with the aortic lumen (Figure 5B). Finally, the patch opening is sutured (Figure 5C) [127]. 

This method does not involve cross-clamp of the aorta and the animals do not suffer cross-clamp-associated damages resulting from a temporary stop of blood supply. Therefore, the method provides for limited interference with the circulation system. This method forms TAAs at the ascending aorta originally [127] and can also apply to the descending thoracic region. The wall of the patch aneurysm is smooth and covered by collagen fibers and endothelium six months after the surgery. This model shows gradual TAA growth, and the TAA diameter increases from 48.9 mm at 3 months to 50.3 at 6 months [127].

This model, together with the pericardium pouch method described in Section 4.4, has a number of limitations. First, there are no elastic fibers and smooth muscle cells on the pericardial patch. Second, the TAA formed are saccular whereas in humans TAA is generally fusiform. Third, the etiology is different from human TAAs. 

### 4.6. Media and Intima Resection

**Figure 6:**
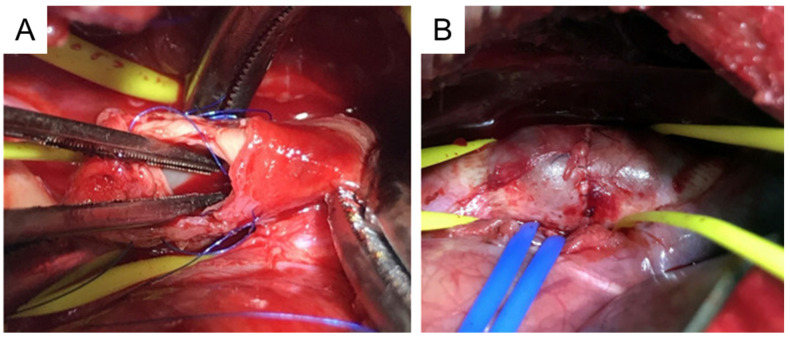
Preparation of porcine TAA using media and intima resection. (**A**), A primary tear is created at the thoracic aorta. (**B**), Aneurysm formation at the thoracic aorta. TAA, thoracic aortic aneurysm. This image is from [121] which was published under the terms of the Creative Commons Attribution Non-Commercial No Derivatives (by-nc-nd) License (http://creativecommons.org/licenses/by-nc-nd/4.0/, accessed on 1 November 2023).

This procedure involves cross-clamping of the aorta. To minimize the impact of cross-clamping, all preparation work is conducted before clamping. This includes left thoracotomy, incising the outer layer of the thoracic aorta, detaching the space under the incised membrane, and suturing both edges of the incised adventitia. After these steps, the thoracic aorta is cross-clamped and the aortic media and intima are resected into a spindle shape of about 10 mm to create a tear (Figure 6A). Next, only the adventitial layer is closed using the sutures that had been placed earlier (Figure 6B). 

The mean time for the whole procedure is ~180 min. The mean aortic clamping time is ~10 min. There is no serious complication associated with the procedure [121]. A saccular TAA is formed in all animals with a mean increase in aortic diameter of 132%.

No patch materials are used, and therefore, the TAAs formed via this method more closely resemble human TAAs compared with the aneurysms created by the previously discussed patch methods. However, this model has a number of limitations. First, it creates a saccular TAA, which is different from a fusiform aortic aneurysm in humans. Second, the TAA formed does not involve atherosclerosis, infiltration of inflammatory cells, and calcification of the aneurysm wall, which are common manifestations of human TAAs.

## 5. Zebrafish TAA Models

Zebrafish have been widely used as a model organism in biological research [128]. Zebrafish contain gene orthologues relating to 70% of total human genes and 82% of morbidity-associated human genes [129], supporting the relevance of this model for human diseases. Zebrafish models have several advantages: zebrafish (a) are low-cost; (b) have transparent embryos that develop ex utero and therefore permit direct microscopic assessment; (c) can be easily genetically modified, with thousands of transgenic mutated strains or fluorescent reporter lines available; (d) are small in size and require less infrastructure and nursing than mammalian models [128]; and (e) are highly suitable for large scale testing and could be used for unbiased small-molecule suppressor screening, as by day 5 post fertilisation, the aneurysm phenotype has 100% penetrance and animals at this stage are small enough to allow screening in a microwell format [130]. 

Zebrafish mutant models are used to assess candidate genes associated with TAA [131]. It has been reported that zebrafish develop TAAs at the outflow tract (equivalent to human aortic root) when they are deficient in latent TGFβ-binding protein 1 and 3 [130] and TGFβ receptor 1 [132]. In addition, wild-type zebrafish could develop TAA when they are treated with TGFβ receptor 1 inhibitor LY364947 [130]. Moreover, zebrafish develop aortic aneurysms in the abdominal region when they are treated with angiotensin II or smoke snuff [133].

A limitation of using the zebrafish TAA model is that TAA is developing very fast, i.e., within within 2–5 days post-fertilisation [130,132]. Thus, zebrafish TAAs may not closely mimic human TAAs which develop over decades.

## 6. Summary of the Animal Models of TAA

The advantages and disadvantages of animal models of TAA are summarized in Table 6.

Section 3, Section 4, Section 5 and Section 6 focus on the animal models of TAA, and the next few Sections (Section 7, Section 8, Section 9 and Section 10) focus on the current views of TAA pathogenesis that are derived from recent studies using these animal models.

## 7. Role of TGFβ in TAA Pathogenesis

**Figure 7:**
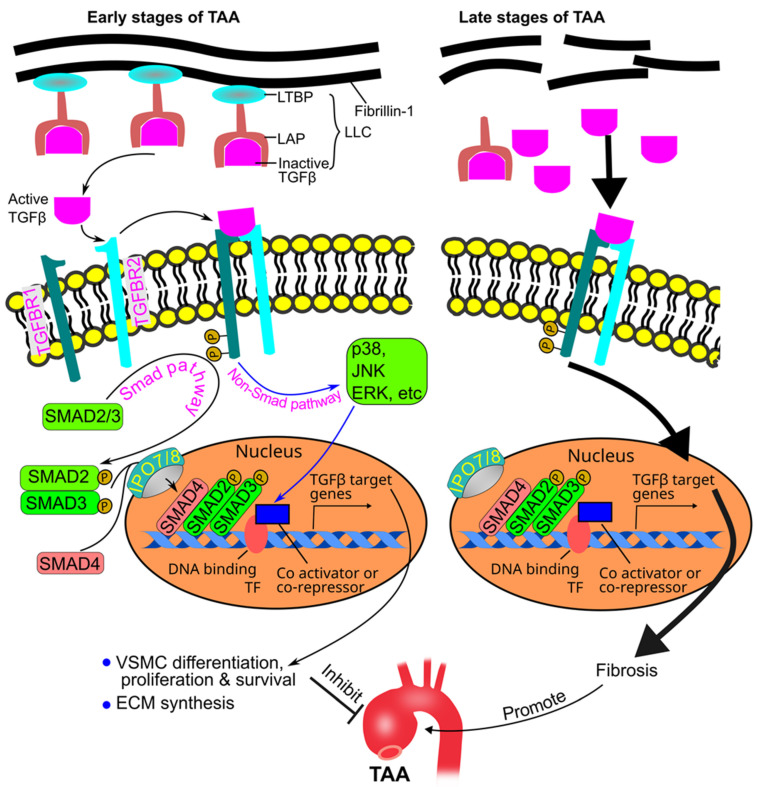
Stage-specific role of TGFβ in TAA pathogenesis. In the early stages of TAA formation, TGFβ binds to its receptors and regulates gene expression through SMAD and non-SMAD pathways, leading to SMC differentiation, proliferation and survival, as well as ECM synthesis. These TGFβ-induced changes inhibit TAA formation. In the late stages of TAA, degradation of the ECM leads to excessive release of active TGFβ and consequent overactivation of TGFβ pathways and thus fibrosis. Excessive fibrosis promotes TAA progression. ECM, extracellular matrix; ERK, extracellular signal–regulated kinase; IPO, importin; Jnk, Jun N-terminal kinase; LAP, latency-associated peptide; LLC, large latent TGFβ complex; LTBP, large latent TGFβ binding protein; SMAD, suppressor of mothers against decapentaplegic; TAA, thoracic aortic aneurysm; TF: transcription factor; TGFβ, transforming growth factor-beta; TGFBR1, TGFβ type 1receptor; TGFBR2, TGFβ type 2 receptor; VSMC, vascular smooth muscle cells.

TGFβ is secreted as large latent complexes which are composed of TGFβ, latency-associated peptide, and latent TGFβ binding protein. Latent TGFβ binding protein anchors the complex to the extracellular matrix fibrillin-1 [134,135] (Figure 7).

TGFβ binds to its TGFβ receptor type 2 (TGFBR2) on the cell surface, and the latter recruits and phosphorylates TGFβ receptor type 1 (TGFBR1). Upon activation, TGFBR1 phosphorylates SMAD proteins (SMAD2 and SMAD3). Phosphorylated SMAD2 and 3 bind to SMAD4 and translocate into the nucleus via importin 7 and 8 [136], where they regulate the expression of TGFβ target genes in association with transcriptional factors and cofactors (co-activators or co-repressors) [24,137,138]. TGFβ can also signal through non-SMAD (noncanonical) pathways, including those mediated by mitogen-activated protein kinases (MAPKs) [21] (Figure 7). The TGFβ pathway protects against abdominal aortic aneurysm [139]. In contrast, early studies supported a hypothesis that the TGFβ pathway promotes TAA formation. Habashi et al. originally reported that administration of a neutralizing TGFβ antibody to 7-week-old *Fbn1*^C1041/+^ mice for 8 weeks inhibited TAA [140]. This hypothesis is also supported by the observation that TGFβ is hyperactive in human aortas with advanced TAAs [141].

However, recent evidence strongly supports an alternative interpretation, i.e., the TGFβ pathway is protective against TAA. Genetic studies have shown that deletion of the TGFβ type 2 receptor in VSMCs promotes TAA and dissection which is associated with increased inflammation, elastolysis, and proteoglycan accumulation [108,109,142,143]. In addition, deletion of *SMAD3* leads to aortic dilation and loss of connections between VSMCs and the elastic fibers, which is accompanied by a reduction in expression of extracellular matrix proteins (e.g., those necessary for assembly and maturation of elastic fibers), integrins, and focal adhesion adaptor proteins [144]. Moreover, deficiency in importin 8, which is required to translocate SMAD proteins into the nucleus [136], promotes TAA formation in mice [145].

TGFβ2 is likely to be responsible for normal aortic development and maintenance of aortic integrity because haploinsufficiency of this ligand causes TAAs in both humans and mice [146,147]. Moreover, pharmacological studies have shown that neutralization of TGFβ signaling starting at postnatal day 16 in *Fbn1*^mgR/mgR^ mice (before TAA formation) accelerates aneurysm formation [148].

These conflicting observations between the earlier and recent observations seem to be due to the difference in the TAA stages under study. Indeed, Cook et al. reported that treating *Fbn1*^mgR/mgR^ mice with TGFβ neutralizing antibodies before aneurysm formation (starting from 16 days after birth) exacerbated TAA formation; however, when the treatment was initiated after aneurysm formation (starting from 45 days after birth), the intervention mitigated TAA development and improved survival [148].

Therefore, the current understanding of the role of TGFβ in TAA is stage-specific: TGFβ protects against TAA at early stages of the disease, and it promotes disease progression at late stages [21]. TGFβ hyperactivity is a secondary driver of maladaptive tissue remodeling in advanced stages of TAAs, and this hyperactivity results from an abnormal increase in active TGFβ due to the degradation of the extracellular matrix (Figure 7).

## 8. Role of the Tunica Intima in TAA Pathogenesis

Abnormal endothelial function has been reported in humans and animals complicated with TAA. Endothelial function is decreased in *Fbn1*^C1041G/+^ mice compared with wild-type littermates as indicated by a decrease in acetylcholine-induced vasorelaxation [149]. In patients with Marfan syndrome, flow-mediated endothelium-dependent vasodilation is impaired [150]. In addition, in mice with BAPN-induced TAA, endothelial tight junctions are dysfunctional, as indicated by an increase in endothelial permeability; on the other hand, the protease-activated receptor 2 inhibitor AT-1001, which improves endothelial tight junction function, decreases TAA incidence [38].

Breeding *Fbn1*^C1041G/+^ mice to a strain expressing a constitutively active eNOS mutant leads to a 46% decrease in aortic root enlargement compared with *Fbn1*^C1041G/+^ mice [151]. In addition, overexpression of eNOS transgene in *Fbn1*^C1041G/+^ mice decreases the aortic root diameter by 58% [151]. Moreover, treating *Fbn1*^C1041G/+^ mice with the eNOS activator caveolin-1 peptide mimetic decreases the aortic root diameter by 60% [151]. Finally, treatment with the NOS inhibitor L-NAME blocks losartan-induced protection against TAA [151]. Therefore, enhanced eNOS-dependent endothelial function protects against TAAs in animals.

## 9. Role of the Tunica Media in TAA Pathogenesis

### 9.1. VSMC-Elastin-Contractile Unit

**Figure 8:**
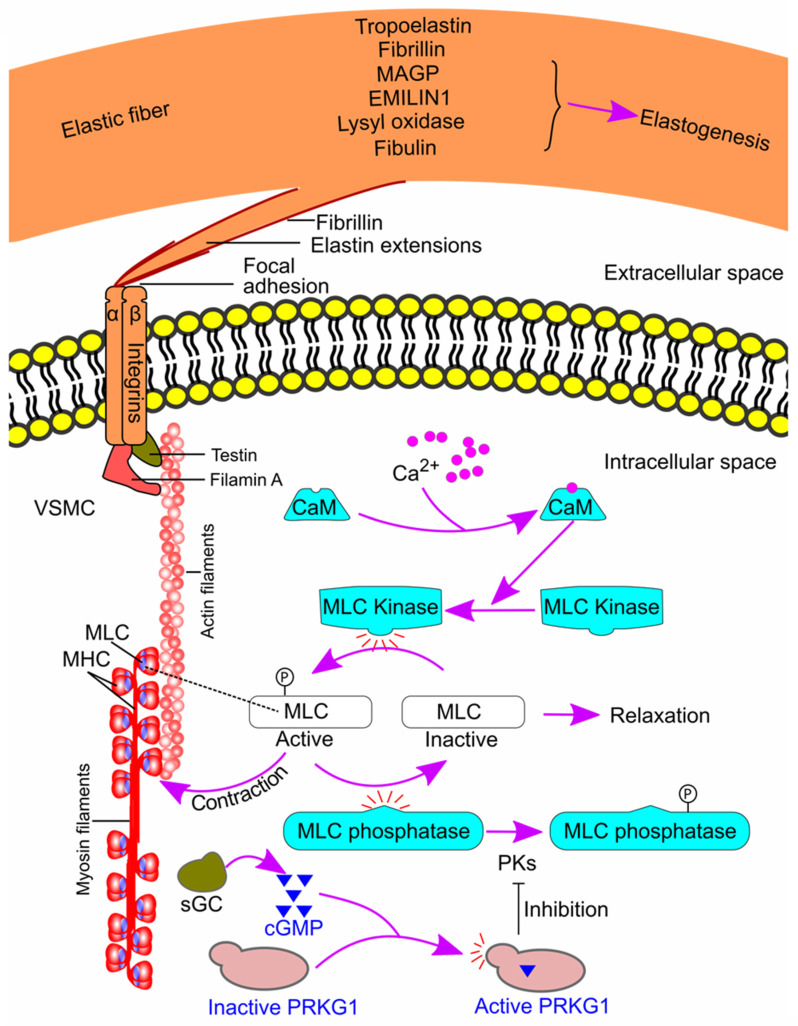
The VSMC-elastin contractile unit. Extensions from the elastin link to integrin receptors on the VSMC surface. The integrin receptors link the contractile actin and myosin filaments inside the cells, thus forming the VSMC-elastin-contractile unit. An increase in intracellular Ca^2+^ activates the contractile unit, leading to contraction. The effect of Ca^2+^ is mediated by calmodulin, MLC kinase, and phosphorylated MLC. On the other hand, activation of sGC leads to relaxation of the contractile unit. The effect of sGC is mediated by cGMP, PRKG1, and MLC phosphatase. CaM, calmodulin; cGMP, cyclic guanosine monophosphate; EMILIN1, elastin microfibril interfacer protein 1; MAGP, microfibril-associated glycoprotein; MHC, myosin heavy chain; MLC, myosin light chain; p, phosphate group; PK, protein kinase; PRKG1, type 1 cGMP-dependent protein kinase; sGC, soluble guanylate cyclase; VSMC, vascular smooth muscle cell.

The VSMC-elastin-contractile unit is a functional and structural unit in the tunica media and plays a vital role in maintaining the structural integrity and function of the thoracic aortic wall. The elastic fibers are organized as a core of elastin surrounded by microfibrils that are composed of fibrillin, microfibril-associated glycoproteins (MAGPs), elastin microfibril interfacer protein 1 (EMILIN1), fibulins, and other glycoproteins [152]. Elastogenesis [153] and the VSMC-elastin contractile unit [152,154,155] were reviewed previously and are summarized in Figure 8. Briefly, extensions from the elastic fibers link to the VSMC surface, and the connection is composed of focal adhesion at the end of elastin extension and integrin receptors on VSMCs. The integrin receptors then link to the contractile actin and myosin filaments inside the cells, forming the VSMC-elastin-contractile unit. An increase in intracellular Ca^2+^ activates the contractile unit. Ca^2+^ binds to calmodulin and activates myosin light chain (MLC) kinase. The latter enzyme then phosphorylates MLC and initiates contraction [156]. On the other hand, the activation of soluble guanylate cyclase results in smooth muscle relaxation via its product cyclic guanosine monophosphate (cGMP). cGMP activates cGMP-dependent protein kinase, which inhibits the deactivation of MLC phosphatase. As a result, active MLC is dephosphorylated and becomes inactive, leading to smooth muscle relaxation [152,154,155] (Figure 8). 

### 9.2. Role of VSMC-Elastin-Contractile Unit Components in TAA Pathogenesis

As the VSMC-elastin-contractile unit is vital in maintaining aortic integrity, defects in the proteins involved in the pathway could lead to TAA formation [24]. Indeed, many proteins are associated with TAAs in humans [155,157,158,159,160,161,162,163,164,165,166,167,168,169,170], and some (fibrillin-1, fibulin-4, microfibril-associated glycoprotein 1/2, lysyl oxidase, testin, thrombospondin domain containing 4, soluble guanylate cyclase, and cGMP-dependent protein kinase) have been confirmed as playing a causal role in TAA formation in animal models [80,88,97,104,105,106,161,165,166,171,172,173,174,175,176] (Table 7).

### 9.3. Role of VSMC Apoptosis in TAA Pathogenesis

VSMC apoptosis has been shown in both human and mouse TAAs [177,178]. VSMC apoptosis impairs the function of the VSMC-elastin-contractile unit and the integrity of the aortic wall [179]. It has been reported that inducing SMC-specific apoptosis in apolipoprotein E-deficient mice fed with a high-fat diet leads to elastic lamina breaks [180], a hallmark of TAA formation. Inhibition of apoptosis using Q-VD-OPh (a pan-caspase inhibitor) decreases TAA formation in *Fbn1*^C1039G/+^ mice [178]. These results support an important role of VSMC apoptosis in TAA pathogenesis.

### 9.4. Role of Inflammation and Reactive Oxygen Species in TAA Pathogenesis

The contribution of inflammation and reactive oxygen species to TAA pathogenesis has been reviewed previously [23,152]. These authors pointed out that increased inflammation and reactive oxygen species lead to VSMC apoptosis and elastin degradation, thus weakening the VSMC-elastin-contractile unit and promoting TAA formation. The role of iNOS (an inflammatory marker) in TAA formation has been highlighted by Oller et al. [181]. They reported that iNOS was increased in TAA samples from patients with Marfan syndrome and *Fbn1*^C1041G/+^ mice. In addition, pharmacological and genetic inhibition of iNOS in *Fbn1*^C1041G/+^ mice decreased TAA diameter to normal levels and regressed elastic fiber fragmentation [181]. The iNOS-induced elastin degradation was associated with an increase in MMP-9 in the aortic medial layer. Consistent with the above report, de la Fuente-Alonso et al. [80] found that inhibition of NO downstream signaling molecules (soluble guanylate cyclase and type 1 cGMP-dependent protein kinase) decreased TAA formation. Another recent example demonstrating the role of inflammation in TAA pathogenesis is IL-6 by Ju et al. [182]. They reported that *IL-6* deficiency inhibited TAA formation in mgR/mgR mice which was associated with a decrease in MMP-9 activity and elastin degradation [182].

### 9.5. Role of Glycosaminoglycans and Proteoglycans in TAA Pathogenesis

An increase in glycosaminoglycans and proteoglycans in the thoracic aorta leads to swelling of the aortic wall and an increase in mechanical stress, consequently triggering weakening of the aortic wall [110]. In addition, an increase in glycosaminoglycans and proteoglycans leads to an increase in immune responses [113] which is accompanied by increased expression of MMP-12 [111].

## 10. Role of the Tunica Adventitia in TAA Pathogenesis

Fibroblasts are the most abundant cell type in the tunica adventitia [183]. They produce the adventitial extracellular matrix, major components of which are types I and III collagens [184]. Mutations in collagen genes of *COL1A2* [185] (encoding collagen I) and *COL3A1* [186] (encoding collagen III) have been identified in Ehlers–Danlos syndrome patients with TAA. Whether a deficiency in collagen I or III promotes TAA formation in research animals needs to be investigated in the future. 

## 11. Potential TAA Treatments Derived from Recent Preclinical Studies 

This section discusses TAA treatment options as they emerge from recent studies using animal models.

### 11.1. Inhibition of Inflammation

Inflammation plays an essential role in TAA formation. Therefore, inhibiting inflammation may protect against TAA. Indeed, many recent studies support this notion (Table S1). Such therapies include treatments with dexamethasone [27,108], folic acid [81], metformin [39], melatonin [31], digoxin [90], oltipraz [33], TEPP-46 (activator of glycolytic enzyme pyruvate kinase M2) [32], cordycepin [65], myriocin [36], senkyunolide I [187], allopurinol [188], angiotensin 1–7 [48], macrophage inhibitors [34], and angiogenic factor with G-patch and FHA domains 1 [40].

In addition, moderate exercise [41], gene therapy using antisense oligonucleotide against angiotensinogen [76], and stem cell therapies [59] are also effective in inhibiting inflammation and TAA formation in animal models.

### 11.2. Inhibition of Apoptosis

VSMC apoptosis is a feature for both human and animal TAAs [177,178]. VSMC apoptosis plays an important role in TAA formation by weakening the VSMC-elastin-contractile unit and the integrity of the aortic wall. Recent studies have shown that diesel exhaust particulate-induced [189], methamphetamine-induced [179], and ciprofloxacin-induced increase in TAA [180] is associated with an increase in VSMC apoptosis. On the other hand, many treatments that inhibit apoptosis also inhibit TAA formation. Such treatments include dexamethasone [27], activators of glycolytic enzyme pyruvate kinase M2 [32], cordycepin [65], nitro-oleic acid [77], and oltipraz [33] (Table S1). The anti-apoptotic effect of these therapies is often associated with their anti-inflammatory properties [27,32,33,65], suggesting that inflammation is an important contributor to the increase in apoptosis seen in TAA tissues.

### 11.3. Inhibition of Elastin Degradation

Elastin is the dominant extracellular matrix component in the tunica media and plays a vital role in maintaining vascular integrity. Elastin degradation leads to the weakening of the aortic wall and thus TAA formation. 

Inhibition of a number of pathways is effective in animals to inhibit elastin degradation and TAA formation (Table S1). Such pathways include the mTOR pathway [28,84,86], the clotting pathway [103], the iNOS-NO-sGC-PRKG1 pathway [80,181], the Notch pathway [91], as well as pathways involving homeodomain-interacting protein kinase 2 [92], androgen receptor [82], and MMPs [29]. In addition, supplementation of the NAD+ precursor nicotinamide riboside to normalize mitochondrial function [52] and gene therapy with microRNA (e.g., AgomiR-22 and miR-133a [53,63]) inhibit elastin degradation and protect against TAA formation.

### 11.4. Other Recent Interventions 

Table S1 also lists a number of other recent interventions that are reported to inhibit or promote TAA development in animal models. For example, administration of vitamin B [87] and baclofen (a GABAB receptor agonist) [93] inhibits TAA development. In addition, inhibition of TGFβ by LY364947 promotes TAA formation [130].

## 12. Conclusions

A large range of mouse models are currently available for TAA research. In recent years, porcine and zebrafish TAA models have been developed. These new TAA models are expected to accelerate TAA research and open the door to pharmacological TAA treatments. Porcine models have an advantage in investigating treatment devices due to the large size of porcine aortas, and zebrafish models have the potential for large-scale small-molecule suppressor screening as this animal model could be applied in a microwell format. A number of potential therapeutic options have been suggested from recent investigations using animal models, and whether they are effective in limiting human TAA growth is expected to be investigated in the future.

## Figures and Tables

**Table 1:** Recent studies employing BAPN-induced TAA models in rodents.

Species, Strain	Sex	Age (w)	BAPN Dose	Induction Time, w	Aortic Diameter Increase	TAA Rate, %	Rupt. Rate,%	Dissection	Ref.
Mice, C57BL/6	M	3	1 g/kg/d in DW	4 w	Yes	NR	NR	Yes	[25]
Mice, C57BL/6	M	3	1 g/kg/d in DW	4 w	Yes	NR	NR	Yes	[26]
Mice, C57BL/6	M	3	1 g/kg/d in DW	4 w	Yes	NR	45.7	Yes	[27]
Mice, C57BL/6	M	3	1 g/kg/d in DW	4 w	Yes	73.3	46.7	Yes	[28]
Mice, C57BL/6	M	3	1 g/kg/d in DW	4 w	Yes	NR	<80	Yes	[29]
Mice, C57BL/6	M	3	1 g/kg/d in DW	4 w	NR	NR	80	Yes	[30]
Mice, C57BL/6	M	3	1 g/kg/d in DW	4 w	Yes	NR	23.3	Yes	[31]
Mice, C57BL/6	NR	3	1 g/kg/d in DW	4 w	Yes	NR	14.3	Yes	[32]
Mice, C57BL/6	NR	3	1 g/kg/d in DW	4 w	Yes	NR	>50	Yes	[33]
Mice, C57BL/10	M	3	0.5 g/kg/d in DW	4 w	NR	NR	<60	Yes	[34]
Mice, C57BL/6	M	3	0.5 g/kg/d in DW	4 w	Yes	NR	11	Yes	[35]
Mice, C57BL/6	M	3	0.5 g/kg/d in DW	4 w	NR	NR	12.5	Yes	[36]
Mice, C57BL/6	M	3	0.5 g/kg/d in DW	1–4 w	Yes	NR	86.7	Yes	[37]
Mice, C57BL/6	F	3	0.5 g/kg/d in DW	1–4 w	Yes	NR	58.8	Yes	[37]
Mice, C57BL/6	M	3	0.4 g/100 g diet	18 d	Yes	NR	NR	Yes	[38]
Mice, C57BL/6	M	3	6 g/L in DW	4 w	Yes	83.3	NR	Yes	[39]
Mice, C57BL/6	NR	3	2.5 g/L in DW	4 w	Yes	42.9	42.9	Yes	[40]
Mice, C57BL/6 SJL	M	3–4	3 g/L in DW	26 w	Yes	50	15.2	NR	[41]
Mice, C57BL/6	M	3	1 g/L in DW	6 w	Yes	NR	66	NR	[42]
Rats, SD	M	3	1 g/kg/d, intragastric	4 w	Yes	16.7	0	NR	[43]

BAPN, β-aminopropionitrile; d, day; DW, drink water; F, female; M, male; NR, not reported; Rupt., rupture; SD, Sprague-Dawley; w, weeks.

**Table 2:** Recent studies employing rodent TAA models induced by angiotensin II infusion.

Species	Strain	Sex	Age, w	Dose, μg/kg per min	Time, w	TAA Rate, %	Rupture Rate, %	Dissection Rate, %	Ref.
Mice	ApoE^−/−^	M	12	1	4	NR	NR	NR	[47]
Mice	ApoE^−/−^	M	8–10	1	4	NR	NR	NR	[48]
Mice	ApoE^−/−^	M	adult	NR	NR	NR	NR	NR	[49]
Mice	WT & Plce1^−/−^	M&F	10 to 12	1	4	80	43	NR	[50]
Mice	WT & Loxl4^−/−^	M	14 or 20	1 or 1.5	4	28.6	NR	NR	[51]
Mice	WT & Tfam^−/−^	M	4–5	1	4	100	70	70%	[52]
Rat	SD	F	8	1.2	4	85	NR	NR	[53]

ApoE^−/−^, apolipoprotein E-deficient; F, female; Loxl4^−/−^, lysyl oxidase (LOX)-like proteins 4-deficient; M, male; NR, not reported; Plce1^−/−^, phospholipase Cε-insufficient; SD, Sprague-Dawley; Tfam^−/−^, mitochondrial transcription factor A-deficient; w, week; WT, wild type.

**Table 3:** Recent studies employing elastase-induced TAA models in rodents.

Species, Strain	Sex	Age, w	PPE Site	PPE Dose	PPE Time, min	Experimental Period, w	TAA Rate, %	Rupture Rate, %	Dissection Rate, %	Ref.
Mice, C57BL/6	M	8–12	ATA & arch	15 µL	5 or 10	1–4	43 or 71	0 or 18	NR	[58]
Mice, C57BL/6	M	8–12	DTA	NR	4	2	NR	NR	NR	[59]
Mice, C57BL/6	M	8–10	DTA	12 μL	3	2	100	NR	NR	[60]
Rats, SD	F	12	DTA	NR	15–20	NR	NR	NR	NR	[61]

ATA, ascending thoracic aorta; DTA, descending thoracic aorta; NR, not reported; PPE, porcine pancreatic elastase; SD, Sprague-Dawley; TAA, thoracic aortic aneurysm; w, week.

**Table 4:** Recent studies employing CaCl_2_-induced TAA models in rodents.

Species, Strain	Sex	Age	CaCl_2_ Conc.	CaCl_2_ Time, min	Exp. Duration, Weeks	Aortic Diameter Increase	TAA Rate, %	Rupt. Rate,%	Dissection Rate, % (or Yes/No)	Ref.
Mice, 129/SvE	M&F	NR	0.5 M	15	4	25%	NR	NR	NR	[62]
Mice, C57BL/6	M&F	10 w	0.5 M	15	4, 8, 16	59.5% 4 w 64.3% 8 w 62.9% 16 w	90%	NR	NR	[63]
Rats, WS	M	NR	0.5 M	15	4	18%	NR	NR	NR	[64]
Rats, SD	NR	NR	0.5 M	15	4	NR	NR	NR	NR	[65]

CalCl_2_, calcium chloride; Conc., concentration; Exp., experimental; F, female; M, male; NR, not reported; Ref, reference; Rupt., rupture; SD, Sprague-Dawley; TAA, thoracic aortic aneurysm; w, week; WS, Wistar.

**Table 5:** Recent studies employing combination of BAPN and angiotensin II-induced TAA models in rodents.

Mous Strain	Sex	Age, w	BAPN			Angiotensin II		TAA Rate, %	TAA Diam ↑	Rup.	Dis.	Ref.
			Dose	Time, Days	Route	Dose, μg/kg/min	Time, Day					
C57BL/6	M	3	0.15 μg/kg/d	28	i.p.	1	3	40	Yes	Yes	NR	[68]
C57BL/6	M	3	0.15 μg/kg/d	28	i.p.	1	28	73	Yes	Yes	NR	[68]
C57BL/6	M & F	10–15	0.2%	28	DW	1	25 (3 days after start of BAPN)	NR	Yes	Yes	NR	[69]
C57BL/6, or FVB	M	3	0.4%	28	Diet	1	1	NR	NR	Yes	Yes	[46]
C57BL/6	M	3	1 μg/kg/d	28	DW	1	2	25	Yes	Yes	Yes	[70]

↑, increase, BAPN, β-aminopropionitrile; d, day; Diam, diameter; Dis., dissection; DW, drinking water; i.p., intraperitoneal; NR, not reported; Ref, reference; Rup., rupture; TAA, thoracic aortic aneurysm; w, week.

**Table 6:** Summary of animal models of TAA.

Species	Models	Features/Advantages	Disadvantages
Mouse/rat	Overall	Low-cost compared with porcine models. Many GMO animals are available	Not suitable for angiographic imaging and testing TAA repair procedures/devices
	BAPN	Showing thrombosis, elastic fiber degradation, VSMC apoptosis, and dissection	May not closely represent human TAA development which requires years/decades
	AngII	A model of thoracoabdominal aortic aneurysms with dissection	As above
	Elastase	TAA location can be controlled	TAAs decrease in size when they reach maximal dilatation at 2 weeks post-surgery
	CaCl_2_	Showing aortic calcification, apoptosis, inflammation, and ECM degradation	This model is not suitable for investigating TAA progression and rupture
	BAPN + AngII	Showing sex phenotype, aortic dissection, intramural hematomas, elastic fiber degradation, and inflammation	May not closely represent human TAA development which requires years/decades
	HFD + AngII	Suitable to study TAA-associated dissection	As above
	TAC	It mimics pressure overload	The increase in aortic diameter is small
	Genetic	*Fbn1*^C1041G/+^ and *Fbn1*^mgR/mgR^ mice are useful for studying Marfan syndrome	Genetic knockout may be lethal
Porcine	Overall	Suitable for angiographic imaging, assessing new devices or interventions	Expensive; Lacking reliable methods to assess anesthetic depth during surgery; GMO pigs are not readily available
	Elastase	Showing loss of VSMCs and degradation of elastic fibers	As above
	Collagenase + CaCl_2_	Showing an increase in MMPs, and a decrease in VSMCs	Not showing rupture
	Vein patch	Valuable in developing novel treatments for endoleaks after TEVAR	TAAs are histologically different from real aneurysms; TAAs are saccular
	Pericardium pouch	Showing mural thrombi and increased inflammation	No elastic fibers and VSMCs on the patch; TAAs formed are saccular; etiology is different from human TAAs
	Cover-then-cut	Showing gradual TAA growth	As above
	MI resection	TAAs more resemble human TAAs than those derived from patch methods	TAAs formed are saccular; they do not involve inflammatory cell infiltration and calcification
Zebrafish	Genetic and pharmacological	Low-cost; less infrastructure requirement; suitable for direct microscopic assessment and large-scale small-molecule suppressor screening in microwells	TAAs develop within 2–5 days and may not closely mimic human TAAs

AngII, angiotensin II; BAPN, β-aminopropionitrile; CaCl_2_, calcium chloride; ECM, extracellular matrix; Fbn1, fibrillin-1; GMO, genetically modified organism; HFD, high-fat diet; MI, media and intima; MMP, matrix metalloproteinase; TAA, thoracic aortic aneurysm; TAC, transverse aortic constriction; TEVAR, thoracic endovascular aortic repair; VSMCs, vascular smooth muscle cells.

**Table 7:** Contribution of proteins in the VSMC-elastin-contractile unit to TAA formation in humans and animals.

Gene	Protein	Associated with TAA in Humans	Contributing to TAA in Animal Models
*FBN1*	Fibrillin-1	Yes [155]	Yes [88,97]
*FBLN4*	Fibulin-4	Yes [157]	Yes [104,105,106]
*MFAP 2* & *5*	Microfibril-associated glycoprotein 1 & 2	Yes [158]	Yes [171]
*LOX*	Lysyl oxidase	Yes [159]	Yes [172]
*TES*	Testin	Yes [160]	Yes [160]
*THSD4*	Thrombospondin, type I, domain containing 4 ^a^	Yes [161]	Yes [161]
*GUCY*	Soluble guanylate cyclase	Yes [162]	Yes [80]
*PRKG1*	Type 1 cGMP-dependent protein kinase	Yes [166]	Yes [80]
*ACTA2*	SMC-specific α actin	Yes [163]	No [173]
*MYH11*	Smooth muscle myosin heavy chain	Yes [164]	No [174]
*MYLK*	Myosin light chain kinase	Yes [165]	No [165]
*EMILIN1*	Elastin microfibril interface–located protein 1	Yes [167]	No [175]
*ELN*	Elastin	Yes [168]	NR [176]
*EMILIN1*	Elastin microfibril interfacer 1	Yes [167,169]	NR [175]
*FLNA*	Filamin A	Yes [170]	NR

NR, not reported. cGMP, cyclic guanosine monophosphate; VSMC, vascular smooth muscle cell; TAA, thoracic aortic aneurysm. ^a^ This protein is a microfibril-associated protein that binds directly to fibrillin-1 and promotes the assembly of the fibrillin-1 matrix.

## Data Availability

Not applicable.

## Supplementary Materials

The supporting information can be downloaded at: https://www.mdpi.com/article/10.3390/ijms25020901/s1.
