# Supplementary material for: Animal Models, Pathogenesis, and Potential Treatment of Thoracic Aortic Aneurysm
Source: Int J Mol Sci. 2024 Jan 11;25(2):901. doi: 10.3390/ijms25020901 (PMC10815651; doi:10.3390/ijms25020901)
Supplement: Supplementary file 1 [file ijms-25-00901-s001.zip › ijms-2804391-supplementary.pdf]

International Journal of *Molecular Sciences*

Supplementary tables

Animal Models, Pathogenesis, and Potential Treatment of Thoracic Aortic Aneurysm

Yutang Wang [1,] *, Indu S. Panicker [1], Jack Anesi [1], Owen Sargisson [1], Benjamin Atchison [1] and Andreas J. R. Habenicht [2]

1 Discipline of Life Science, Institute of Innovation, Science and Sustainability, Federation University Australia, Ballarat, VIC 3353, Australia; indu.p@federation.edu.au (I.S.P.); jackanesi@students.federation.edu.au (J.A.); owensargisson@students.federation.edu.au (O.S.); benjaminatchison@students.federation.edu.au (B.A.)
2 Institute for Cardiovascular Prevention, Ludwig-Maximilians-Universität München (LMU), 80336 Munich, Germany; andreas.habenicht@med.uni-muenchen.de
* Correspondence: yutang.wang@federation.edu.au

International Journal of *Molecular Sciences*

MDPI

Table S1. Recent studies investigating potent therapeutic target against TAA in animal models

TAA induction	Animals	Intervention	TAA incidence	Aortic diameter	Rupture	Mechanism	Targets	Ref
Inflammation								
BAPN	C57BL/6 mice	Dexamethasone	↓	↓	NR	↓ Macrophage and neutrophil infiltration<br>↓ Apoptosis of VSMC<br>↓ MMP 2/9<br>↓ ECM degradation	Inflammation<br>Apoptosis<br>ECM degradation	[1]
Genetic	VSMC-specific Tgfbr2-deficient mice	Dexamethasone	↓	↓	NR	↓ CCL8<br>↓ Macrophage infiltration<br>↓ MMP2<br>↓ NF-κB	Inflammation	[2]
Genetic	Fbn1$^{C1039G/+}$ mice	Folic acid	↓	↓	NR	↓ NOX4<br>↓ Superoxide production<br>↓ Elastin fiber fragmentation	Inflammation<br>ECM degradation	[3]
BAPN	C57BL/6	Metformin	↔	↓	↔	↓ Inflammation<br>↓ Elastin breakage	Inflammation<br>ECM degradation	[4]
BAPN	C57BL/6 mice	Melatonin	↓	↓	↓	↑ SIRT1 signalling<br>↓ Macrophage infiltration<br>↓ MMP2, MMP9<br>↓ Reactive oxygen species<br>↓ VSMC loss	Inflammation<br>Oxidative stress	[5]
Genetic	Fbn1$^{mgR/mgR}$	Digoxin	NR	↓	NR	↑ miR-122,<br>↓ CCL2	Inflammation<br>ECM degradation	[6]

						↓ MMP12<br>↓ Elastin fragmentation		
BAPN	C57BL/6	Oltipraz (Nrf activator)	↓	↓	↓	↓ Apoptosis<br>↓ Macrophage infiltration<br>↓ MMP	Inflammation<br>Apoptosis	[7]
BAPN	C57BL/6 mice	TEPP-46 (activator of glycolytic enzyme pyruvate kinase M2)	↓	↓	↓	↓ Inflammatory cells infiltration<br>↓ ROS<br>↓ Caspase 1<br>↓ VSMCs loss	Inflammation<br>Oxidative stress<br>Apoptosis	[8]
$CaCl_2$	Sprague-Dawley rats	Cordycepin, (an anti-inflammatory and antioxidant compound)	↓	↓	NR	↓ VEGF<br>↓ IL-6, TNF-α and IL-1β<br>↓ ROS<br>↓ Caspase 3/9 and apoptosis	Inflammation<br>Oxidative stress<br>Apoptosis	[9]
BAPN	C57Bl/6 mice	Myriocin	↓	NR	↓	↓ Inflammation (IL-1β, TNF-α, and IL-6)	Inflammation	[10]
BAPN+ AngII	C57Bl/6 mice	Senkyunolide I	↓	↓	↓	↓ Inflammation<br>↓ ROS<br>↓ Apoptosis<br>↑ Elastin integrity	Inflammation<br>Oxidative stress<br>Apoptosis<br>ECM degradation	[11]
Ang II, BAPN, Genetic	Mice	Allopurinol	↓	↓	↓	↓ Uric acid<br>↓ Inflammation	Inflammation	[12]
Ang II	$ApoE^{-/-}$ mice	Angiotensin 1-7	↓	↓	NR	↓ Inflammation<br>↓ MMP2, 9<br>↑ Elastin integrity	Inflammation	[13]
BAPN	C57BL/10 mice	Macrophage inhibitors Ki20227, mLR12	↓	↓	↓	↓ Macrophage infiltration<br>↓ Inflammation	Inflammation	[14]

						↓ MMP 2&9		
BAPN<br>TAC<br>Genetic	C57B/L6 mice<br>Fbn1$^{C1041G/+}$ mice	Angiogenic factor with G-patch and FHA domains 1	↓	↓	NR	↓TGF-β and ERK1/2<br>↓ Inflammation	TGFβ<br>Inflammation	[15]
BAPN	C57/BL6<br>SJL	Moderate aerobic exercise	↓	↓	↓	↓ TGF-β pathway<br>↓ Inflammatory markers<br>↑ Elastogenesis	Inflammation<br>ECM formation	[16]
Genetic	Fbn1$^{C1039G/+}$ mice	Antisense oligonucleotide against angiotensinogen	↓	↓	NA	↓ Inflammatory gene expression<br>↓ Elastin fragmentation	Inflammation<br>ECM degradation	[17]
Elastase	C57Bl/6 mice	Administration of mesenchymal stem cells	↓	↓	NR	↓ T cell, neutrophil and macrophage infiltration<br>↓ Proinflammatory cytokines<br>↑ Anti-inflammatory IL-10<br>↓ Elastic degradation	Inflammation<br>ECM degradation	[18]
Apoptosis								
BAPN	SD rats	Methamphetamine	↑	↑	↑	↑ MMP 2&9<br>↑ Elastin breakage<br>↑ VSMC apoptosis	Apoptosis<br>ECM degradation	[19]
BAPN	C57BL/6 Mice	Ciprofloxacin (antibiotic)	↑	↑	↑	↑ Apoptosis<br>↑ MMP9<br>↑ ECM degradation	Apoptosis<br>ECM degradation	[20]
BAPN	C57BL6 mice	Diesel exhaust particulate	↑	↑	↔	↑ apoptosis<br>↑ BAX/Bcl2<br>↑ Caspase 3/cleaved Cas3	Apoptosis	[21]
BAPN	C57BL/6 mice	Dexamethasone	↓	↓	NR	↓ Macrophage and neutrophil infiltration	Inflammation<br>Apoptosis	[1]

						↓ Apoptosis of VSMC<br>↓ MMP 2/9<br>↓ ECM degradation	ECM degradation	
BAPN	C57BL/6 mice	TEPP-46 (activator of glycolytic enzyme pyruvate kinase M2)	↓	↓	↓	↓ Inflammatory cells infiltration<br>↓ ROS<br>↓ Caspase 1<br>↓ VSMCs loss	Inflammation<br>Oxidative stress<br>Apoptosis	[8]
$CaCl_2$	SD rats	Cordycepin, (an anti-inflammatory and antioxidant compound)	↓	↓	NR	↓ VEGF<br>↓ IL-6, TNF-α and IL-1β<br>↓ ROS<br>↓ Caspase 3/9 and apoptosis	Inflammation<br>Oxidative stress<br>Apoptosis	[9]
Genetic	$Fbn1^{C1041G/+}$ mice	Nitro-oleic acid	↓	↓	NR	↓ ERK1/2<br>↓ Smad2<br>↑ NF-κB<br>↓ MMP2<br>↓ Apoptosis	TGFβ<br>Inflammation<br>Apoptosis	[22]
BAPN	C57BL/6	Oltipraz (Nrf activator)	↓	↓	↓	↓ Apoptosis<br>↓ Macrophage infiltration<br>↓ MMP	Inflammation<br>Apoptosis	[7]
ECM degradation								
Genetic	$Fbn1^{C1039G/+}$ mice	Rapamycin	↓	↓	NR	↓miR-126-3p and subsequent ERK1/2 signalling<br>↓MMP-9 expression<br>↓Elastin degradation	ECM degradation	[23]
BAPN	C57BL/6 mice	Rapamycin	↓	↓	↔	↓ mTOR pathway<br>↓ Macrophage and neutrophil infiltration<br>↓ MMP9	mTOR<br>ECM degradation	[24]

						↓ Elastic fiber fragmentation		
Genetic	Mice deficient in hamartin, an inhibitor of mTOR	Rapamycin	↓	NR	NR	↓ mTOR activation<br>↓ Elastic fiber fragmentation	mTOR<br>ECM degradation	[25]
Genetic	Fbln4^{SMKO} C57BL/6 mice	Dabigatran (thrombin inhibitor)<br>Rivaroxaban (factor Xa inhibitor)	↓	NR	NR	↓ Protease activated receptor 1	ECM	[26]
Genetic	Fbn1$^{C1039G/+}$ mice	ODQ (sGC inhibitor)<br>KT5823 (PRKG inhibitor)<br>PRKG1 silencing	NR	↓	NR	↓Elastin fiber fragmentation	NO–sGC–PRKG<br>ECM degradation	[27]
Genetic	Fbn1$^{mgR/mgR}$ mice	DAPT (Notch inhibitor)	NR	↓	↓	↓ Elastin degradation	ECM degradation	[28]
Genetic	Fbn1$^{C1039G/+}$ mice	HIPK2 Inhibitor BT173	↓	↓	↓	↓ Elastin fiber fragmentation<br>↓Collagen accumulation	ECM degradation	[29]
Genetic	Fbn1$^{C1039G/+}$ mice	Flutamide (androgen receptor blocker)	NR	↓	NR	↓ Erk1/2, Smad2<br>↓ MMP2<br>↓ Elastin fiber fragmentation	TGFβ<br>ECM degradation	[30]
BAPN	C57BL/6 mice	Crocin (MMP inhibitor)	↓	↓	↓	↓ MMP activity<br>↓ Elastin degradation	ECM degradation	[31]
Genetic	Fbn1$^{C1039G/+}$ mice	Nicotinamide riboside (NAD+ precursor to normalize mitochondrial function)	NR	↓	NR	↑ Mitochondrial dysfunction<br>↓ Elastin fiber fragmentation	Mitochondrial function<br>ECM degradation	[32]
Ang II	SD rats	AgomiR-22	NR	↓	NR	↓ MMP-9	ECM degradation	[33]

						↑ ECM integrity		
$CaCl_2$	C57BL/6 mice	miR-133a overexpression	↓	↓	NR	↓ Pro – protein convertase furin<br>↓ Elastic fiber fragmentation	ECM degradation	[34]
Miscellaneous								
Genetic	Fbn1$^{C1039G/+}$ mice	Vitamin B	↓	↓	NR	↑Smad4<br>↑ collagen maturation	Collagen maturation	[35]
Genetic	Fbn1$^{mgR/mgR}$ mice	baclofen ($GABA_B$ receptor agonist)	↓	↓	↓	↑ muscle contractility<br>↑ aortic wall microarchitecture	Contractility	[36]
TGFβ inhibition	Zebrafish	TGFβ antagonist LY364947	↑	↑	NR	↓ pSmad3	TGFβ	[37]

↔, no effect; ↑, increase; ↓, decrease; Ang II, angiotensin II; ApoE$^{-/-}$, apolipoprotein E-deficient; BAPN, β-aminopropionitrile; Bcl2, B-cell lymphoma 2; $CaCl_2$, calcium chloride; CCL, chemokine (C-C motif) ligand; ECM, extracellular matrix; Erk, extracellular signal–regulated kinase; Fbn1, fibrillin-1; Fbln4, fibulin-4; Fbln4^{SMKO}, smooth muscle-specific fibulin-4 knockout; GABA, gamma-aminobutyric acid; HIPK2, homeodomain-interacting protein kinase 2; IL, interleukin; MMP, matrix metalloproteinases; mTOR, mammalian target of rapamycin; NF-κB, Nuclear Factor Kappa B;NO, nitirc oxide; NR, not reported; Nrf: nuclear factor erythroid 2-related factor 2; Ltbp, latent TGFβ-binding protein; NOX4, NADPH oxidase 4;PRKG1, type 1 cGMP-dependent protein kinase; Ref, reference; ROS, reactive oxygen species; SD, Sprague-Dawley; sGC, soluble guanylate cyclase; SIRT1, sirtuin 1; Smad, suppressor of mothers against decapentaplegic; TGFβ, transforming growth factor-beta; Tgfbr2, TGFβ type 2receptor; TNF-α, tumor necrosis factor alpha; VEGF, vascular endothelial growth factor; VSMCs, vascular smooth muscle cells.

1. Wang, X.; Zhang, X.; Qiu, T.; Yang, Y.; Li, Q.; Zhang, X., Dexamethasone reduces the formation of thoracic aortic aneurysm and dissection in a murine model. *Exp. Cell Res.* **2021,** 405, (2), 112703.
2. Wang, X.; Li, Q.; Li, W.; Zhang, T.; Li, X.; Jiao, Y.; Zhang, X.; Jiang, J.; Zhang, X.; Zhang, X., Dexamethasone attenuated thoracic aortic aneurysm and dissection in vascular smooth muscle cell Tgfbr2-disrupted mice with CCL8 suppression. *Exp. Physiol.* **2022,** 107, (6), 631-645.
3. Huang, K.; Wang, Y.; Siu, K. L.; Zhang, Y.; Cai, H., Targeting feed-forward signaling of TGFβ/NOX4/DHFR/eNOS uncoupling/TGFβ axis with anti-TGFβ and folic acid attenuates formation of aortic aneurysms: Novel mechanisms and therapeutics. *Redox Biol* **2021,** 38, 101757.
4. Ma, W.; Zhang, J.; Liu, S.; Yan, S.; Xu, K.; Zhang, Y. S.; Abudupataer, M.; Ming, Y.; Zhu, S.; Xiang, B.; Zhou, X.; Luo, S.; Huang, H.; Tang, Y.; Zhang, S.; Xie, Z.; Chen, N.; Sun, X.; Li, J.; Lai, H.; Wang, C.; Zhu, K.; Zhang, W., Patient-derived microphysiological model identifies the therapeutic potential of metformin for thoracic aortic aneurysm. *EBioMedicine* **2022,** 81, 104080.
5. Xia, L.; Sun, C.; Zhu, H.; Zhai, M.; Zhang, L.; Jiang, L.; Hou, P.; Li, J.; Li, K.; Liu, Z.; Li, B.; Wang, X.; Yi, W.; Liang, H.; Jin, Z.; Yang, J.; Yi, D.; Liu, J.; Yu, S.; Duan, W., Melatonin protects against thoracic aortic aneurysm and dissection through SIRT1-dependent regulation of oxidative stress and vascular smooth muscle cell loss. *J. Pineal Res.* **2020,** 69, (1), e12661.
6. Zhang, R. M.; Tiedemann, K.; Muthu, M. L.; Dinesh, N. E. H.; Komarova, S.; Ramkhelawon, B.; Reinhardt, D. P., Fibrillin-1-regulated miR-122 has a critical role in thoracic aortic aneurysm formation. *Cell Mol Life Sci* **2022,** 79, (6), 314.
7. Wang, D.; Wu, J.; Le, S.; Wang, H.; Luo, J.; Li, R.; Chen, X.; Song, Y.; Wu, L.; Ye, P.; Du, X.; Huang, X., Oltipraz, the activator of nuclear factor erythroid 2-related factor 2 (Nrf2), protects against the formation of BAPN-induced aneurysms and dissection of the thoracic aorta in mice by inhibiting activation of the ROS-mediated NLRP3 inflammasome. *Eur. J. Pharmacol.* **2022,** 936, 175361.
8. Le, S.; Zhang, H.; Huang, X.; Chen, S.; Wu, J.; Chen, S.; Ding, X.; Chen, S.; Zhao, J.; Xu, H.; Cui, J.; Zou, Y.; Yu, J.; Jiang, L.; Wu, J.; Ye, P.; Xia, J., PKM2 Activator TEPP-46 Attenuates Thoracic Aortic Aneurysm and Dissection by Inhibiting NLRP3 Inflammasome-Mediated IL-1β Secretion. *J. Cardiovasc. Pharmacol. Ther.* **2020,** 25, (4), 364-376.
9. Zhou, M.; Zha, Z.; Zheng, Z.; Pan, Y., Cordycepin suppresses vascular inflammation, apoptosis and oxidative stress of arterial smooth muscle cell in thoracic aortic aneurysm with VEGF inhibition. *Int. Immunopharmacol.* **2023,** 116, 109759.
10. Yang, H.; Yang, F.; Luo, M.; Chen, Q.; Liu, X.; Zhang, Y.; Zhu, G.; Chen, W.; Li, T.; Shu, C.; Zhou, Z., Metabolomic Profile Reveals That Ceramide Metabolic Disturbance Plays an Important Role in Thoracic Aortic Dissection. *Front Cardiovasc Med* **2022,** 9, 826861.
11. Zhao, K.; Zhu, H.; He, X.; Du, P.; Liang, T.; Sun, Y.; Jing, Z.; Zhou, J., Senkyunolide I ameliorates thoracic aortic aneurysm and dissection in mice via inhibiting the oxidative stress and apoptosis of endothelial cells. *Biochim Biophys Acta Mol Basis Dis* **2023,** 1869, (7), 166819.
12. Yang, L.; Wu, H.; Luo, C.; Zhao, Y.; Dai, R.; Li, Z.; Zhang, X.; Gong, Z.; Cai, Z.; Shen, Y.; Yu, F.; Li, W.; Zhao, H.; Zhang, T.; Zhu, J.; Fu, Y.; Wang, J.; Kong, W., Urate-Lowering Therapy Inhibits Thoracic Aortic Aneurysm and Dissection Formation in Mice. *Arterioscler. Thromb. Vasc. Biol.* **2023,** 43, (6), e172-e189.

13. Jadli, A. S.; Ballasy, N. N.; Gomes, K. P.; Mackay, C. D. A.; Meechem, M.; Wijesuriya, T. M.; Belke, D.; Thompson, J.; Fedak, P. W. M.; Patel, V. B., Attenuation of Smooth Muscle Cell Phenotypic Switching by Angiotensin 1-7 Protects against Thoracic Aortic Aneurysm. *Int. J. Mol. Sci.* **2022,** 23, (24).
14. Liu, X.; Chen, W.; Zhu, G.; Yang, H.; Li, W.; Luo, M.; Shu, C.; Zhou, Z., Single-cell RNA sequencing identifies an Il1rn(+)/Trem1(+) macrophage subpopulation as a cellular target for mitigating the progression of thoracic aortic aneurysm and dissection. *Cell Discov* **2022,** 8, (1), 11.
15. Da, X.; Li, Z.; Huang, X.; He, Z.; Yu, Y.; Tian, T.; Xu, C.; Yao, Y.; Wang, Q. K., AGGF1 therapy inhibits thoracic aortic aneurysms by enhancing integrin α7-mediated inhibition of TGF-β1 maturation and ERK1/2 signaling. *Nat Commun* **2023,** 14, (1), 2265.
16. Aicher, B. O.; Zhang, J.; Muratoglu, S. C.; Galisteo, R.; Arai, A. L.; Gray, V. L.; Lal, B. K.; Strickland, D. K.; Ucuzian, A. A., Moderate aerobic exercise prevents matrix degradation and death in a mouse model of aortic dissection and aneurysm. *Am J Physiol Heart Circ Physiol* **2021,** 320, (5), H1786-h1801.
17. Chen, J. Z.; Sawada, H.; Ye, D.; Katsumata, Y.; Kukida, M.; Ohno-Urabe, S.; Moorleghen, J. J.; Franklin, M. K.; Howatt, D. A.; Sheppard, M. B.; Mullick, A. E.; Lu, H. S.; Daugherty, A., Deletion of AT1a (Angiotensin II Type 1a) Receptor or Inhibition of Angiotensinogen Synthesis Attenuates Thoracic Aortopathies in Fibrillin1(C1041G/+) Mice. *Arterioscler. Thromb. Vasc. Biol.* **2021,** 41, (10), 2538-2550.
18. Hawkins, R. B.; Salmon, M.; Su, G.; Lu, G.; Leroy, V.; Bontha, S. V.; Mas, V. R.; Jr, G. R. U.; Ailawadi, G.; Sharma, A. K., Mesenchymal Stem Cells Alter MicroRNA Expression and Attenuate Thoracic Aortic Aneurysm Formation. *J. Surg. Res.* **2021,** 268, 221-231.
19. Luo, B. Y.; Zhou, J.; Guo, D.; Yang, Q.; Tian, Q.; Cai, D. P.; Zhou, R. M.; Xu, Z. Z.; Wang, H. J.; Chen, S. Y.; Xie, W. B., Methamphetamine induces thoracic aortic aneurysm/dissection through C/EBPβ. *Biochim Biophys Acta Mol Basis Dis* **2022,** 1868, (9), 166447.
20. Xiang, B.; Abudupataer, M.; Liu, G.; Zhou, X.; Liu, D.; Zhu, S.; Ming, Y.; Yin, X.; Yan, S.; Sun, Y.; Lai, H.; Wang, C.; Li, J.; Zhu, K., Ciprofloxacin exacerbates dysfunction of smooth muscle cells in a microphysiological model of thoracic aortic aneurysm. *JCI Insight* **2023,** 8, (2), e161729.
21. Ming, Y.; Zhou, X.; Liu, G.; Abudupataer, M.; Zhu, S.; Xiang, B.; Yin, X.; Lai, H.; Sun, Y.; Wang, C.; Li, J.; Zhu, K., PM2.5 exposure exacerbates mice thoracic aortic aneurysm and dissection by inducing smooth muscle cell apoptosis via the MAPK pathway. *Chemosphere* **2023,** 313, 137500.
22. Nettersheim, F. S.; Lemties, J.; Braumann, S.; Geißen, S.; Bokredenghel, S.; Nies, R.; Hof, A.; Winkels, H.; Freeman, B. A.; Klinke, A.; Rudolph, V.; Baldus, S.; Mehrkens, D.; Mollenhauer, M.; Adam, M., Nitro-oleic acid reduces thoracic aortic aneurysm progression in a mouse model of Marfan syndrome. *Cardiovasc. Res.* **2022,** 118, (9), 2211-2225.
23. Liu, M.; Li, L.; Zhu, J.; He, C.; Xu, Q.; Sun, A.; Kong, W.; Li, W.; Zhang, X., Rapamycin attenuates a murine model of thoracic aortic aneurysm by downregulating the miR-126-3p mediated activation of MAPK/ERK signalling pathway. *Biochem Biophys Res Commun* **2019,** 512, (3), 498-504.
24. Zhou, B.; Li, W.; Zhao, G.; Yu, B.; Ma, B.; Liu, Z.; Xie, N.; Fu, Y.; Gong, Z.; Dai, R.; Zhang, X.; Kong, W., Rapamycin prevents thoracic aortic aneurysm and dissection in mice. *J. Vasc. Surg.* **2019,** 69, (3), 921-932.e3.
25. Li, G.; Wang, M.; Caulk, A. W.; Cilfone, N. A.; Gujja, S.; Qin, L.; Chen, P. Y.; Chen, Z.; Yousef, S.; Jiao, Y.; He, C.; Jiang, B.; Korneva, A.; Bersi, M. R.; Wang, G.; Liu, X.; Mehta, S.; Geirsson, A.; Gulcher, J. R.; Chittenden, T. W.; Simons, M.; Humphrey, J. D.; Tellides, G., Chronic mTOR activation induces a degradative smooth muscle cell phenotype. *J. Clin. Invest.* **2020,** 130, (3), 1233-1251.

26. Shin, S. J.; Hang, H. T.; Thang, B. Q.; Shimoda, T.; Sakamoto, H.; Osaka, M.; Hiramatsu, Y.; Yamashiro, Y.; Yanagisawa, H., Role of PAR1-Egr1 in the Initiation of Thoracic Aortic Aneurysm in Fbln4-Deficient Mice. *Arterioscler. Thromb. Vasc. Biol.* **2020,** 40, (8), 1905-1917.
27. de la Fuente-Alonso, A.; Toral, M.; Alfayate, A.; Ruiz-Rodríguez, M. J.; Bonzón-Kulichenko, E.; Teixido-Tura, G.; Martínez-Martínez, S.; Méndez-Olivares, M. J.; López-Maderuelo, D.; González-Valdés, I.; Garcia-Izquierdo, E.; Mingo, S.; Martín, C. E.; Muiño-Mosquera, L.; De Backer, J.; Nistal, J. F.; Forteza, A.; Evangelista, A.; Vázquez, J.; Campanero, M. R.; Redondo, J. M., Aortic disease in Marfan syndrome is caused by overactivation of sGC-PRKG signaling by NO. *Nat Commun* **2021,** 12, (1), 2628.
28. Jespersen, K.; Li, C.; Batra, R.; Stephenson, C. A.; Harding, P.; Sestak, K.; Foley, R. T.; Greene, H.; Meisinger, T.; Cook, J. R.; Baxter, B. T.; Xiong, W., Impact of Notch3 Activation on Aortic Aneurysm Development in Marfan Syndrome. *J Immunol Res* **2022,** 2022, 7538649.
29. Caescu, C. I.; Hansen, J.; Crockett, B.; Xiao, W.; Arnaud, P.; Spronck, B.; Weinberg, A.; Hashimoto, T.; Murtada, S. I.; Borkar, R.; Gallo, J. M.; Jondeau, G.; Boileau, C.; Humphrey, J. D.; He, J. C.; Iyengar, R.; Ramirez, F., Inhibition of HIPK2 Alleviates Thoracic Aortic Disease in Mice With Progressively Severe Marfan Syndrome. *Arterioscler. Thromb. Vasc. Biol.* **2021,** 41, (9), 2483-2493.
30. Tashima, Y.; He, H.; Cui, J. Z.; Pedroza, A. J.; Nakamura, K.; Yokoyama, N.; Iosef, C.; Burdon, G.; Koyano, T.; Yamaguchi, A.; Fischbein, M. P., Androgens Accentuate TGF-β Dependent Erk/Smad Activation During Thoracic Aortic Aneurysm Formation in Marfan Syndrome Male Mice. *J. Am. Heart. Assoc.* **2020,** 9, (20), e015773.
31. Qi, F.; Liu, Y.; Zhang, K.; Zhang, Y.; Xu, K.; Zhou, M.; Zhao, H.; Zhu, S.; Chen, J.; Li, P.; Du, J., Artificial Intelligence Uncovers Natural MMP Inhibitor Crocin as a Potential Treatment of Thoracic Aortic Aneurysm and Dissection. *Front Cardiovasc Med* **2022,** 9, 871486.
32. Oller, J.; Gabandé-Rodríguez, E.; Ruiz-Rodríguez, M. J.; Desdín-Micó, G.; Aranda, J. F.; Rodrigues-Diez, R.; Ballesteros-Martínez, C.; Blanco, E. M.; Roldan-Montero, R.; Acuña, P.; Forteza Gil, A.; Martín-López, C. E.; Nistal, J. F.; Lino Cardenas, C. L.; Lindsay, M. E.; Martín-Ventura, J. L.; Briones, A. M.; Redondo, J. M.; Mittelbrunn, M., Extracellular Tuning of Mitochondrial Respiration Leads to Aortic Aneurysm. *Circulation* **2021,** 143, (21), 2091-2109.
33. Zhao, H. M.; Jin, L.; Liu, Y.; Hong, X., Changes in expressions of miR-22-3p and MMP-9 in rats with thoracic aortic aneurysm and their significance. *Eur. Rev. Med. Pharmacol. Sci.* **2020,** 24, (12), 6949-6954.
34. Akerman, A. W.; Collins, E. N.; Peterson, A. R.; Collins, L. B.; Harrison, J. K.; DeVaughn, A.; Townsend, J. M.; Vanbuskirk, R. L.; Riopedre-Maqueira, J.; Reyes, A.; Oh, J. E.; Raybuck, C. M.; Jones, J. A.; Ikonomidis, J. S., miR-133a Replacement Attenuates Thoracic Aortic Aneurysm in Mice. *J. Am. Heart. Assoc.* **2021,** 10, (16), e019862.
35. Huang, T. H.; Chang, H. H.; Guo, Y. R.; Chang, W. C.; Chen, Y. F., Vitamin B Mitigates Thoracic Aortic Dilation in Marfan Syndrome Mice by Restoring the Canonical TGF-β Pathway. *Int. J. Mol. Sci.* **2021,** 22, (21).
36. Hansen, J.; Galatioto, J.; Caescu, C. I.; Arnaud, P.; Calizo, R. C.; Spronck, B.; Murtada, S. I.; Borkar, R.; Weinberg, A.; Azeloglu, E. U.; Bintanel-Morcillo, M.; Gallo, J. M.; Humphrey, J. D.; Jondeau, G.; Boileau, C.; Ramirez, F.; Iyengar, R., Systems pharmacology-based integration of human and mouse data for drug repurposing to treat thoracic aneurysms. *JCI Insight* **2019,** 4, (11), e127652.
37. Abrial, M.; Basu, S.; Huang, M.; Butty, V.; Schwertner, A.; Jeffrey, S.; Jordan, D.; Burns, C. E.; Burns, C. G., Latent TGFβ-binding proteins 1 and 3 protect the larval zebrafish outflow tract from aneurysmal dilatation. *Dis. Model. Mech.* **2022,** 15, (3), dmm046979.
